# A simple method for *in vivo* labelling of infiltrating leukocytes in the mouse retina using indocyanine green dye

**DOI:** 10.1242/dmm.019018

**Published:** 2015-11-01

**Authors:** Dawn A. Sim, Colin J. Chu, Senthil Selvam, Michael B. Powner, Sidath Liyanage, David A. Copland, Pearse A. Keane, Adnan Tufail, Catherine A. Egan, James W. B. Bainbridge, Richard W. Lee, Andrew D. Dick, Marcus Fruttiger

**Affiliations:** 1 NIHR Biomedical Research Centre for Ophthalmology, Moorfields Eye Hospital NHS Foundation Trust, London EC1V 2PD, UK; 2 University College London, Institute of Ophthalmology, London EC1V 9EL, UK; 3 Academic Unit of Ophthalmology, School of Clinical Sciences, University of Bristol, Bristol BS8 1TD, UK

**Keywords:** Indocyanine green, Inflammation, *In vivo* imaging

## Abstract

We have developed a method to label and image myeloid cells infiltrating the mouse retina and choroid *in vivo*, using a single depot injection of indocyanine green dye (ICG). This was demonstrated using the following ocular models of inflammation and angiogenesis: endotoxin-induced uveitis, experimental autoimmune uveoretinitis and laser-induced choroidal neovascularization model. A near-infrared scanning ophthalmoscope was used for *in vivo* imaging of the eye, and flow cytometry was used on blood and spleen to assess the number and phenotype of labelled cells. ICG was administered 72 h before the induction of inflammation to ensure clearance from the systemic circulation. We found that *in vivo* intravenous administration failed to label any leukocytes, whereas depot injection, either intraperitoneal or subcutaneous, was successful in labelling leukocytes infiltrating into the retina. Progression of inflammation in the retina could be traced over a period of 14 days following a single depot injection of ICG. Additionally, bright-field microscopy, spectrophotometry and flow cytometric analysis suggest that the predominant population of cells stained by ICG are circulating myeloid cells. The translation of this approach into clinical practice would enable visualization of immune cells *in situ*. This will not only provide a greater understanding of pathogenesis, monitoring and assessment of therapy in many human ocular diseases but might also open the ability to image immunity live for neurodegenerative disorders, cardiovascular disease and systemic immune-mediated disorders.

## Abstract (precis)

**Summary:** We show here that peripheral leukocytes can be labelled with ICG *in vivo* and then directly imaged as they invade the retina after inflammatory stimuli.

## INTRODUCTION

Resident and infiltrating leukocytes have an important role in sight-threatening diseases of the eye. Substantial evidence exists to confirm not only that activation and altered immune responses happen in uveitic syndromes (Lee et al., 2014) but that they may also occur in the form of parainflammation (Xu et al., 2009) and initiate or exacerbate age-related macular degeneration and diabetic retinopathy (Whitcup et al., 2013). Here, the cellular participants of the immune system act as drivers for pathological angiogenesis, forming the two main causes of blindness in Western industrialized countries, namely, age-related macular degeneration and diabetic retinopathy (Penfold et al., 1987; Lopez et al., 1991; Lutty et al., 1997; Joussen et al., 2001). A better understanding of the role of inflammation in the development of pathological angiogenesis is essential for the development of disease-modifying strategies not only for monitoring disease progression but also to assess the efficacy of treatments.

The development of clinically viable imaging tools for *in vivo* visualization of inflammation is a crucial step in this process. To date, live imaging of inflammation has been restricted to experimental animal models, with examples such as magnetic particles (iron oxide and gadolinium chelates) for magnetic resonance imaging, fluorescent nanoparticles which can be tagged with aptamers or peptides targeted against cell surface biomarkers, circulating factors or nucleic acid structures, or dyes such as acridine orange, which is a known human carcinogen (Joussen et al., 2001; Rausch et al., 2001; Montet-Abou et al., 2010; Roivainen et al., 2012; Cibiel et al., 2012; Nahrendorf et al., 2007; Hossain et al., 1998). The only application that is currently used to image inflammatory cells in humans is based on *in vitro* radionucleotide labelling of leukocytes from the patient's own blood. Although this allows direct visualization of cell migration patterns, current imaging techniques do not allow sufficient resolution to track single cells (van Hemert et al., 2007).

Here, we present the use of indocyanine green (ICG) for *in vivo* visualization of myeloid cells in the mouse. ICG is a near-infrared (NIR) fluorescence tricarbocyanine dye with a peak spectral absorption of 800-810 nm (in blood) that is approved by the US Food and Drug Administration for clinical use. The introduction of ICG in ophthalmic angiography in the late 1960s was largely because of its minimal toxicity and favourable optical and biophysical properties (Kogure et al., 1970). NIR light could penetrate the ocular pigments of the eye, such as melanin and xanthophyll, thereby allowing visualization of the deep choroidal vasculature (not possible with conventional fluorescein angiography). In addition, its tendency for conjugation to plasma proteins meant that the dye did not readily leak from the fenestrated choriocapillaris (Cherrick et al., 1960).

Despite the wide use of intravenous (i.v.) ICG, US Food and Drug Administration approval and a good safety profile, there is limited evidence for its use in cellular imaging. This is probably because of its pharmacokinetic properties; ICG is rapidly removed from the circulation via hepatic clearance and it has a half-life of approximately 3-4 min, which precludes *in vivo* labelling of cells. However, we observed that by administering ICG as a depot injection, we could obtain reproducible labelling of peripheral CD11b^+^ circulating myeloid cells, establishing a novel method for *in vivo* tracking of these cells at near single-cell resolution as they invade the eye in response to inflammation and injury. Furthermore, we found *in vitro* evidence that human myeloid cells stain in a similar manner, which strongly supports the translation of this promising technique into clinical practice.

**TRANSLATIONAL IMPACT**

**Clinical issue**

Inflammation underpins most of the pathological consequences of human disease. Until now, measurement of inflammation has been confined to surrogate markers, such as the size of a lesion, its appearance, or the levels of inflammatory mediators in the blood. Resident and infiltrating leukocytes are important players in eye diseases, and a better understanding of their role in these conditions is essential not only for monitoring disease progression but also to assess the efficacy of treatments. The development of clinically viable imaging tools for *in vivo* visualization of inflammation is a crucial step in this process, but current imaging techniques do not allow sufficient resolution to track single cells.

**Results**

In this work, the authors describe a simple method by which cellular effectors of inflammation – namely, white-blood cells – can be visualized, measured, and monitored over time in the mouse retina. They use a single depot injection of indocyanine green (a dye that has been used safely in humans for blood flow measurements for more than 60 years) to label inflammatory cells in the periphery. This allowed them to track the invasion of those cells into the retina in eye disease models. Retinal infiltration of inflammatory cells was detected in mice in three models of retinal inflammation and angiogenesis, after a single depot injection of indocyanine green dye. Cells could be monitored in the retina for a period of 14 days, and the large majority were identified as infiltrating lymphatic and myeloid cells.

**Implications and future directions**

If translated successfully into humans, this method will allow direct monitoring of inflammation and assessment of treatment strategies in a variety of diseases, including not only human ocular diseases but also neurodegenerative disorders, cardiovascular diseases and systemic immune-mediated disorders.

## RESULTS

### *In vitro* labelling of peripheral blood mononuclear cells (PBMCs) and splenocytes with ICG

**Fig. 1.**
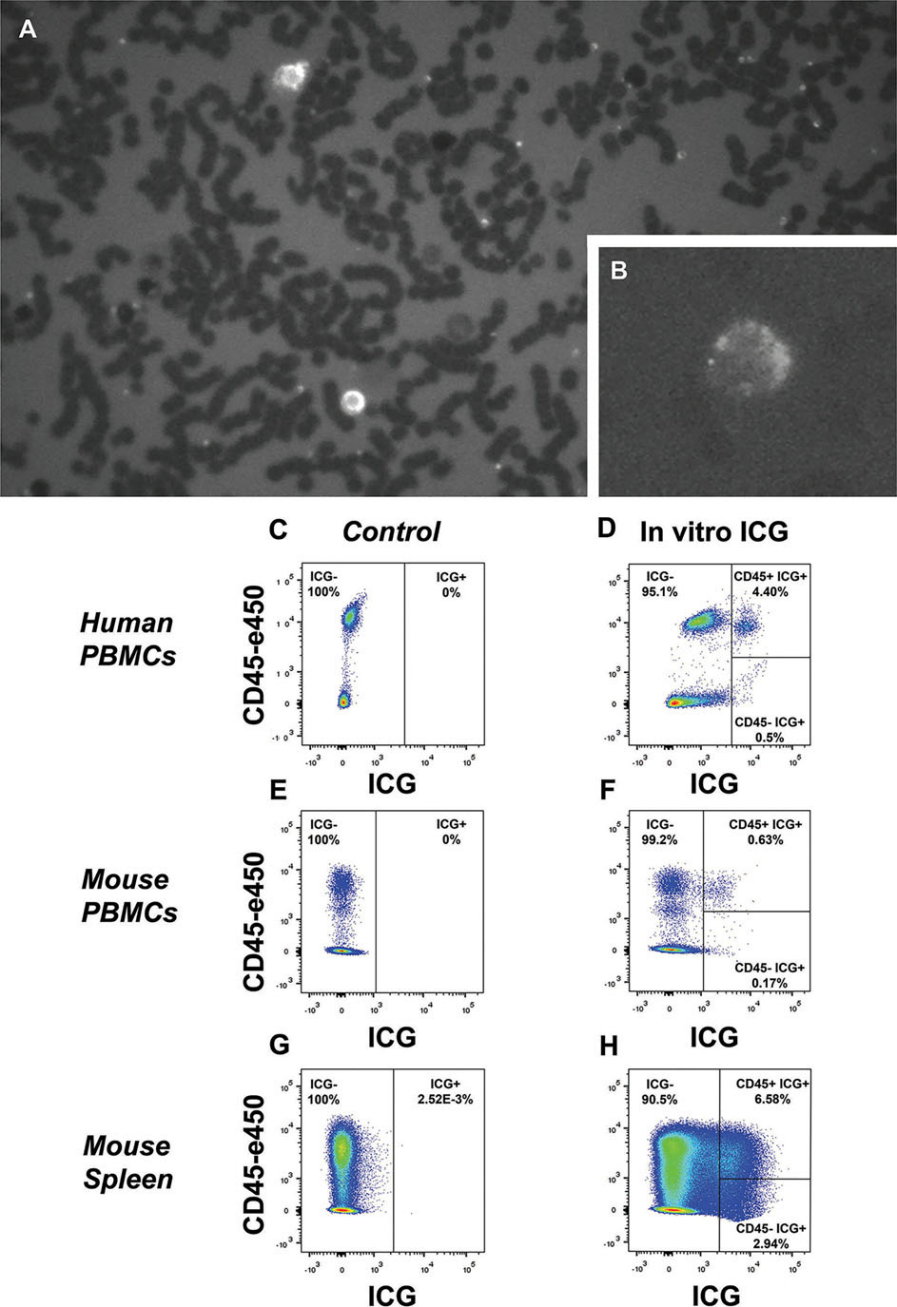
***In vitro* labelling of peripheral blood mononuclear cells (PBMCs) and splenocytes in humans and mice.** (A,B) Blood smear of human blood incubated in a concentration of 6.25 µg/ml indocyanine green dye (ICG) for 30 min at room temperature. A small proportion of cells stained with ICG were visualized on the near-infrared channel at 10× magnification (A) and 20× magnification (B). (C,D) Detection of ICG-stained human PBMCs by flow cytometry in cells incubated in PBS and 6.25 µg/ml of ICG reveals that 4.9% of all cells were labelled and, of these, 4.4% were CD45-high. (E,F) In mouse PBMCs, although ICG labelling was also observed in the same *in vitro* conditions, a smaller proportion of ICG-labelled cells were detected; 0.8% of total cells. (G,H) Mouse splenocytes were more readily labelled with ICG, with 9.5% of total cells staining with ICG.

To establish whether ICG can label circulating leukocytes, we incubated whole blood with the dye *in vitro* for 30 min at room temperature. Visual inspection of a blood smear from human blood by fluorescent microscopy revealed a small population of fluorescent cells in the NIR channel (Fig. 1A,B). Next, we exposed PBMCs isolated from human and mouse blood, and mouse splenocytes to ICG (30 min at room temperature) and then analysed dye uptake and cell identity by flow cytometry. In human PBMCs, whilst all cells stained slightly with the dye, around 2-5% of all cells were strongly stained with ICG (Fig. 1C,D). In mouse PBMCs, <1% of all cells were strongly stained with ICG (Fig. 1E,F) compared with 7-10% of mouse splenocytes, which consist of both splenic reservoir monocytes and resident macrophages (Fig. 1G,H).

We observed that although the proportion of ICG-stained cells increased when incubated at higher temperatures and in higher concentrations of ICG, there was an increase in non-specific staining. Furthermore, the staining of cells varied with the number of washes and volume of media used. We identified that a 30 min incubation of ICG at a concentration of 6.25 µg/ml at room temperature, with two washes in 5 ml PBS, achieved reproducible staining of cells. Furthermore, we identified that the minimal dose at which stained cells could be detected was 1.5 µg/ml, although 5 µg/ml was required for reliable and reproducible staining. *In vitro*, the specificity of ICG binding to PBMCs was dependent upon the concentration, ambient temperature and period of incubation (data not shown).

### Assessment of the efficiency of ICG cell labelling and its effects on macrophages and lymphocytes

**Fig. 2.**
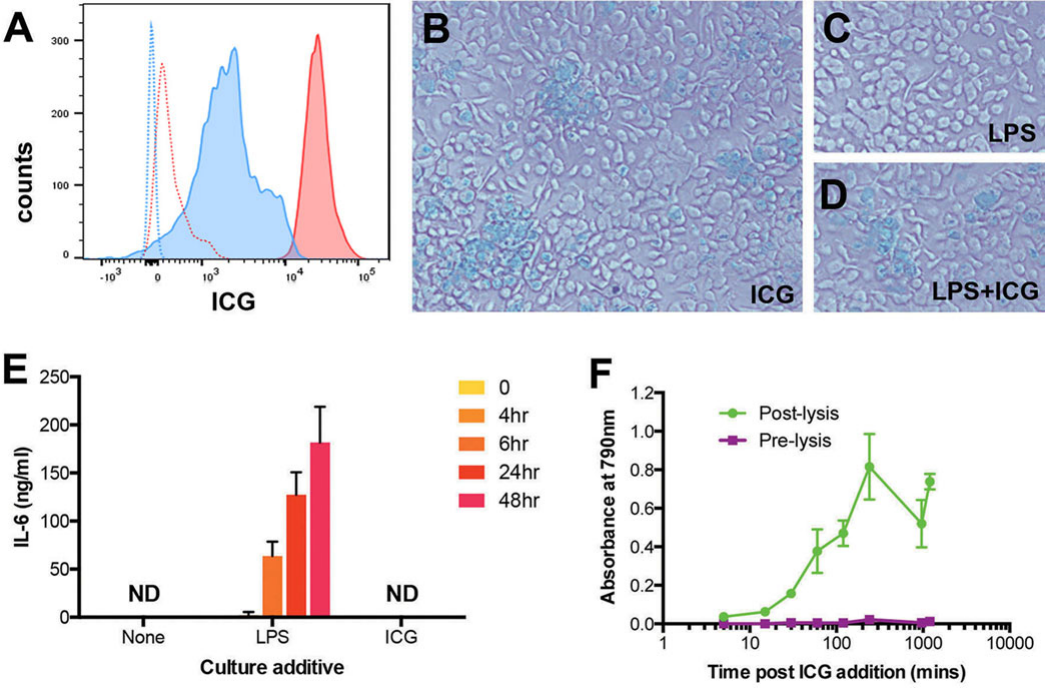
**ICG labels macrophages by internalization of the dye but does not appear to cause activation.** (A) *In vitro*-labelled bone-marrow-derived macrophages (BMDMs, red) appear in flow cytometry more strongly labelled than CD4^+^ lymphocytes (blue; unlabelled cells are indicated by stippled lines). (B) Bright-field microscopy of BMDMs cultured for 2 h with 0.2 mg/ml ICG identifies regions of visible green dye apparently within cells. (C,D) Applying LPS, 5 ng/ml LPS only (C) and LPS+ICG (D) did not lead to a marked difference in ICG uptake. (E) There is no evidence of classical activation, as measured by the production of interleukin-6 (IL-6) from supernatants taken at different time points. Data were combined from two separate experiments (means+s.d. are shown). (F) BMDMs were incubated with 0.2 mg/ml ICG. Following several washes, PBS was incubated with the cells for 5 min, then tested by spectrophotometry and compared with supernatants from the same cells after lysis with 2% Triton. Increasing the duration of ICG incubation led to increased absorption, consistent with the release of progressive internalized ICG.

To assess the efficiency of ICG uptake of different populations of cells, we used cultured mouse bone-marrow-derived macrophages (BMDMs) and magnetic-activated cell sorted CD4^+^ mouse lymphocytes from the spleen. We observed, using flow cytometry, that whilst both populations were stained with ICG, BMDMs were more strongly stained than CD4^+^ cells (Fig. 2A). A 2 h exposure of BMDMs to ICG (200 ng/ml) led to a green stain of BMDMs that was visible in bright-field microscopy (Fig. 2B). The uptake of ICG in BMDMs was not affected by activation of the cells with lipopolysaccharide (LPS; Fig. 2C,D). Conversely, ICG did not activate BMDMs, as assessed by interleukin-6 (IL-6) production (Fig. 2E). In order to assess whether ICG was internalized by the cells or whether it only stained the cell membrane, we measured ICG release of stained cells after lysis by spectrophotometry. Increasing absorption at 790 nm suggested the release of internalized dye (Fig. 2F).

### *In vivo* labelling of infiltrating leukocytes by ICG

First, we examined whether ICG labelling was possible *in vivo*, and second, whether these labelled cells could also be imaged *in vivo*. To this end, we administered 1 mg of ICG by intraperitoneal (i.p.) injection to C57BL/6 mice 3 days before they were imaged in three disease models in which there is substantial leukocytic (including monocyte/macrophage) retinal infiltration.

**Fig. 3.**
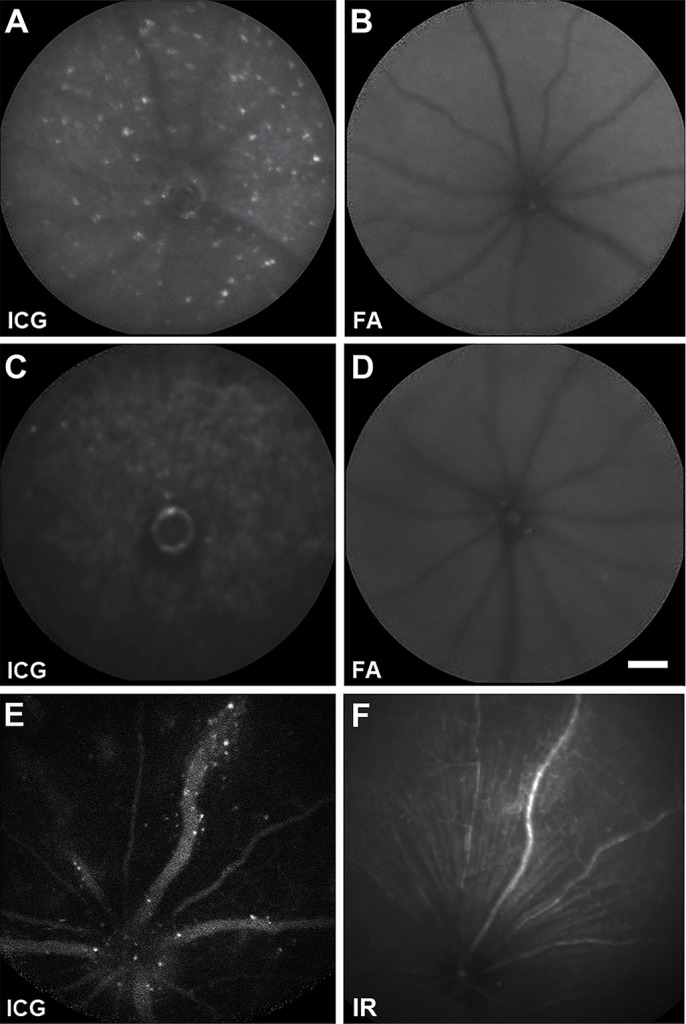
***In vivo* labelling of infiltrating leukocytes in two murine models of ocular inflammation.** (A) Inflammatory infiltration of the deep retina/choroid of an eye with endotoxin-induced uveitis, imaged using a scanning laser ophthalmoscope with a near-infrared filter (790-nm diode excitation laser and 800-nm long-pass filter). ICG-labelled cells were visualized as white dots throughout the 55° field of view after an intraperitoneal (i.p.) injection of ICG (5 days before imaging) and induction of systemic inflammation with an i.p. injection of lipopolysaccharide (2 days before imaging). (B) An image of the deep retina/choroid of the same mouse was taken in the fluorescein angiography (AF) channel, using a blue-light filter (488-nm solid-state excitation laser and 500-nm long-pass filter). No white dots are present, demonstrating that white dots imaged in A are not a consequence of autofluorescence but ICG-labelled cells. (C) A control mouse that only received i.p. ICG (3 days before imaging). Imaging with a near-infrared filter showed only a few sporadic ICG-labelled cells, suggesting a low-level circulation of myeloid cells into the retina. (D) An image of the deep retina/choroid of the same mouse was obtained using the 488-nm channel, illustrating that the identified cells were not autofluorescent in this range. (E) Inflammation of a retinal vein (vasculitis) is visualized using the near-infrared filter in an eye at peak experimental autoimmune uveitis. ICG-labelled cells were visualized as white dots clustering around a segment of vasculitis. Mice with experimental autoimmune uveitis received an injection of i.p. ICG 3 days before imaging peak disease on day 26. (F) An infrared-reflectance (IR) image (820-diode excitation laser, no barrier filter) of the same mouse was obtained, which demonstrates the segment of retinal vein affected by vasculitis with increased reflectance (higher white intensity) of the vein itself, and surrounding tissues. Of note, no white dots were observed in this image, indicating that again, the white dots observed in (C) are not the result of autofluorescence but of ICG-labelled cells.

The first model was endotoxin-induced uveitis induced by systemic delivery of LPS from *Escherichia coli*. A previous study detected Acridine Orange-labelled leukocytes in the subretinal space/deep retina, 2 days after LPS injection (Miyahara et al., 2004). We found that ICG-labelled cells were detectable in the retina *in vivo* using fluorescence scanning laser ophthalmoscopy, 24 h after LPS injection, with a peak at 2 days after LPS administration (and 5 days after i.p. ICG; Fig. 3A). No signal was seen in the fluorescein channel (488 nm solid-state excitation laser and 500 nm barrier filter), indicating that the ICG signal did not derive from autofluorescence (Fig. 3B). Control animals that were injected only with ICG but not with LPS showed only a few sporadic ICG^+^ cells (Fig. 3C,D). This suggests low levels of peripheral cell trafficking in the normal retina, as previously inferred via flow cytometric analysis (Boldison et al., 2014; Chu et al., 2013), which dramatically increases after LPS stimulation.

The second model was experimental autoimmune uveoretinitis, an antigen-dependent CD4^+^ T-cell-initiated ocular autoimmune disease, which shares features with human uveitis. Unlike the endotoxin-induced uveitis model, which produces a diffuse, short-lived inflammation in the deep retina and choroid, the prominent features of the experimental autoimmune uveoretinitis model include marked infiltration of myeloid and T-cells around inner retinal blood vessels (vasculitis) and a more sustained inflammation with retinal infiltration and damage and remodelling developing over many weeks (Dick et al., 1996). We found that 26 days after immunization of these animals, ICG-labelled cells could be visualized in the retina, choroid and particularly in areas of active vasculitis (Fig. 3E,F), correlating with published histology of activated myeloid cells in this model (Dick et al., 1995).

**Fig. 4.**
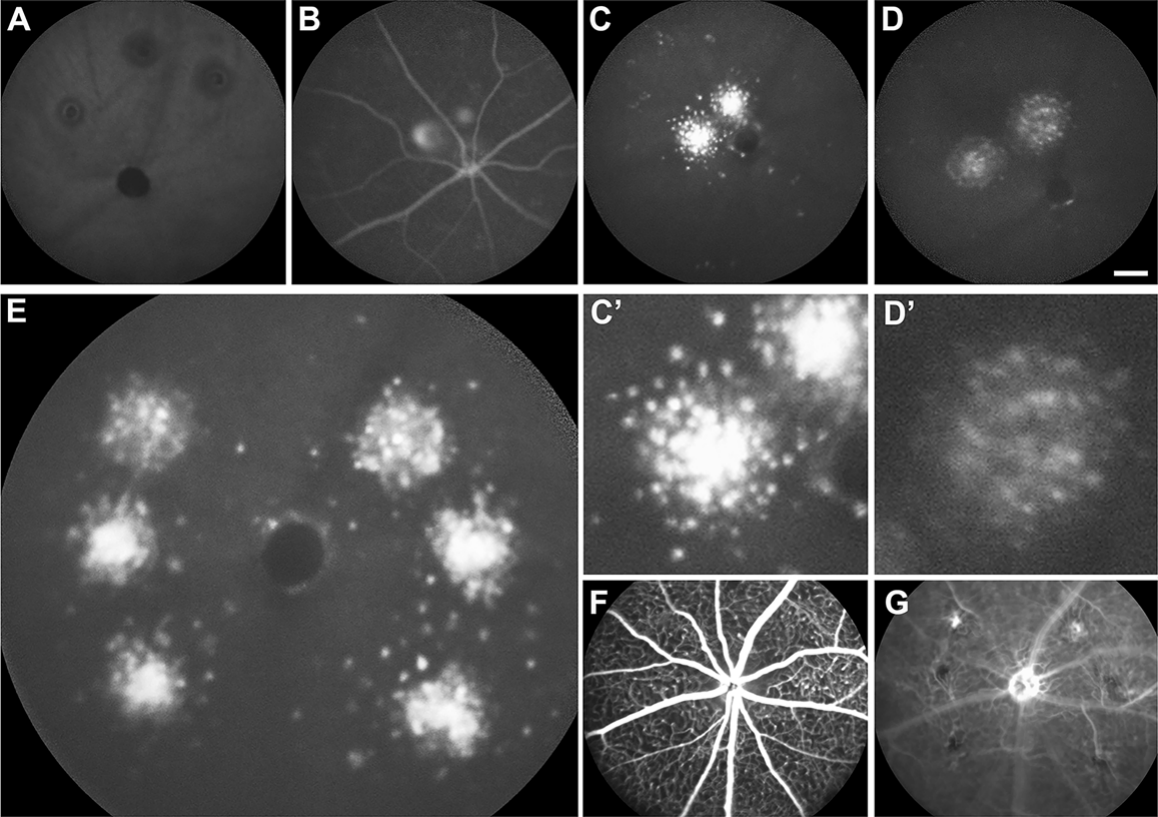
***In vivo* labelling of infiltrating leukocytes in a laser-induced choroidal neovascularization (CNV) murine model.** The scanning laser ophthalmoscope with a near-infrared filter (790-nm diode excitation laser and 800-nm long-pass filter) was used to image the retina and choroid. (A) A deep retinal/choroidal image of a control animal that did not receive an i.p. injection of ICG. No fluorescence was detected using the near-infrared filter. (B) A deep retinal/choroidal image of an animal that did receive i.p. ICG and laser induction of CNV, showing fluorescence in the retinal vessels and laser lesions. No obvious leakage of ICG could be seen in the surrounding retinal tissues. (C) A deep retinal/choroidal image of an animal that received i.p. ICG (10 days before imaging) and laser induction of CNV (7 days before imaging), showing an accumulation of ICG-labelled cells in and around the laser lesions. (C′) Magnified image of laser lesion and surrounding cells. (D) A deep retinal/choroidal image of the same animal shown in C 3 days later (13 days after i.p. ICG), showing that the intensity of the ICG signal has reduced. (D′) Magnified image of laser lesion and surrounding cells. (E) A deep retinal/choroidal image of an animal that received i.p. ICG (10 days before imaging) with six laser-induced CNV lesions (7 days before imaging), showing accumulation of ICG-labelled cells in and around all six laser lesions. (F,G) Corresponding fluorescein angiography images of the superficial retina and deep retina/choroid obtained 10 min after i.p. injection with 100 µl of fluorescein dye and imaged with a blue-light filter (488-nm solid-state excitation laser and 500-nm long-pass filter). (G) Deep retinal/choroidal images show that only two of six laser lesions subsequently developed choroidal neovascularization, and none of these 1-week-old lesions showed obvious fluorescein dye leakage into the surrounding tissues, suggesting that inflammatory cellular infiltration occurred independently of vascular leakage.

The third model was the laser-induced choroidal neovascularization (CNV) model, a model widely used to study pathological angiogenesis in the retina (Montezuma et al., 2009). The vascular response in this model is accompanied by an accumulation of infiltrating leukocytes in the laser lesion from the circulation and from within the retina (Horie et al., 2013; Liu et al., 2013). We hypothesized that the infiltrating leukocytes can be visualized by labelling cells with ICG in the periphery. Given that laser injury can induce autofluorescence, we first imaged animals without ICG. In the NIR channel, no fluorescence was detected in the lesion (Fig. 4A). Next, we tested whether administration (i.p.) of ICG immediately after the laser would result in ICG leakage into the retina. Although ICG could be visualized in the retinal vasculature and laser lesions, overt leakage of ICG into the retina was not apparent (Fig. 4B). As before, in order to ensure that there was sufficient time for ICG labelling of cells in the circulation and the clearance of ICG from the circulation, we injected ICG 3 days before laser induction of CNV. Given that ICG is rapidly cleared from the circulation, we reasoned that a 3-day delay would be sufficient to minimize any ICG in the circulation that might leak directly into the retina from the bloodstream and label resident retinal macrophages. Consistent with this, ICG could no longer be detected in the circulation by NIR imaging at this stage (not shown). Seven days after induction of the laser lesion (10 days after ICG), we observed a marked aggregation of clearly labelled cells in and around the laser lesions (Fig. 4C,C′). After a further 3 days (10 days after laser), the signal started to subside (Fig. 4D,D′). ICG^+^ cells were observed in laser lesions that developed CNV and also in those that had no fluorescein angiographic evidence of CNV after 7 days. This is illustrated by an example shown in Fig. 4E. Here, six laser lesions were applied. Although all of them accumulated ICG^+^ cells (Fig. 4E), only two lesions showed signs of neovascularization based on fluorescein angiography (Fig. 4F,G).

Furthermore, dissection of the mice 7 days after administration of ICG i.p. revealed green staining of lymphatic tissue in the thoracic cavity (Fig. S1A,B), mediastinal lymph nodes (Fig. S1C), thymus (not shown) and the greater omentum in the abdominal cavity (Fig. S1D-F). This suggests that inflammatory cells in the circulation, most probably both monocytes and lymphocytes that continuously circulate between the bloodstream and lymphoid organs, are labelled by the ICG. To check whether ICG might be toxic to the cells that take it up, we used flow cytometry to measure cell death in inflammatory cells that had been stained *in vitro* (after 30 min) and *in vivo* (10 days after ICG injection) in the CNV model. This showed no noticeable differences in cell counts, indicating that ICG has no gross effects on inflammatory cell populations in the spleen, the retina and the choroid (Fig. S2) within the time frame of our experiments (10 days).

**Fig. 5.**
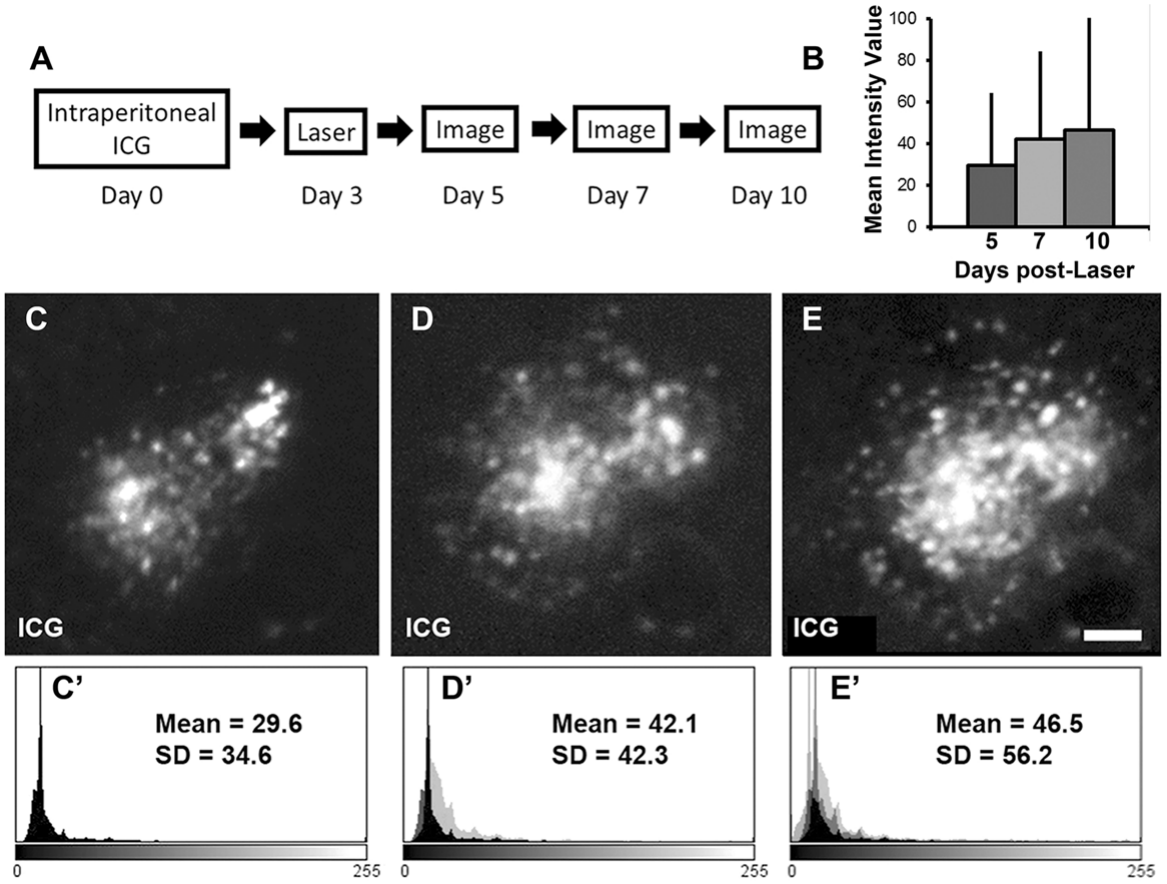
***In vivo* quantification of laser-induced CNV-related infiltration.** (A) ICG was injected i.p. at day 0 before laser induction of CNV at day 3 in C57BL/6 mice. Imaging with the scanning laser ophthalmoscope using a near-infrared filter (790-nm diode excitation laser and 800-nm barrier filter) was performed on days 5, 7 and 10. (C-E) Increasing cellular infiltration in and surrounding the laser-CNV lesion was observed over time. (B,C′-E′) We demonstrate that the mean intensity values and standard deviations can be quantified over a given area and show an increase in inflammation over time. The mean intensity value of the lesion at day 5 is represented by the black histogram (C′), day 7 the superimposed light grey histogram (D′) and day 10 the superimposed medium grey histogram (E′).

We next tested whether cellular infiltration around the laser-induced CNV lesion could be monitored over time. As before, ICG was administered i.p. 3 days before the laser, and animals were imaged sequentially 2, 5 and 8 days after laser CNV induction (Fig. 5). The number of ICG-labelled cells in and surrounding the CNV lesion could be observed qualitatively to increase over time (Fig. 5C-E). Next, we assessed the feasibility with which this could be quantified reproducibly. First, we attempted a simple pixel count by thresholding individual CNV lesions (Fig. S3); a method we have used successfully for quantifying ICG-labelled cells in endotoxin-induced uveitis models (data not shown). However, because of the narrow range of intensity values within each lesion, it was not possible to visualize individual cells overlying the CNV lesion. Nonetheless, we found that by assessing the mean intensity values for a given pixel area using histograms, we were able to quantify cellular infiltration over CNV lesions (Fig. S3G). Using this method, we demonstrated that ICG-labelled cellular infiltration can be quantified and monitored over time (Fig. 5B,C′-E′).

Given that ICG is routinely used in clinical practice, we compared the dosage and delivery routes normally used in humans (5 mg i.v. bolus injection) with our mouse protocol (1 mg i.p. bolus injection). We reduced the ICG in a stepwise fashion from 1 mg to 0.0125 mg, which roughly equates to the human dose in ophthalmic use. Invading cells could still be detected readily after administration of 0.5 mg ICG i.p., but at 0.25 mg they were fainter. With progressively reduced amounts of ICG (0.125, 0.05, 0.025 and 0.0125 mg), the lesions were faintly fluorescent, but individual cells could no longer be detected (Fig. S4). Out of the different delivery routes tested, i.p. was the most efficient at labelling cells. A subcutaneous depot (1 mg) produced weaker labelling, but individual cells could still be detected, whereas i.v. (1 mg) delivery produced only very faint staining. In addition, i.v. delivery of ICG shortly before the laser produced only staining of the retinal and choroidal vasculature, with diffuse leakage of ICG into the laser-induced CNV lesion, and individual cells could not be detected (not shown). Oral administration (by gavage or in drinking water) did not lead to any labelling (Fig. S5).

### Characterization of *in vivo* ICG-labelled cells

**Fig. 6.**
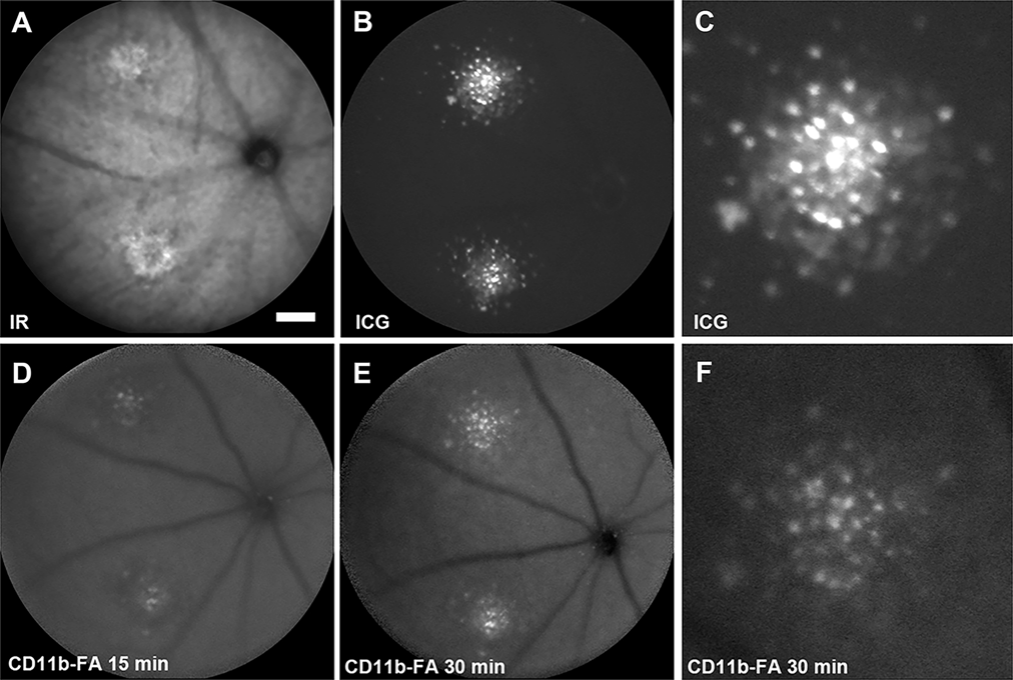
**Characterization of *in vivo* ICG-labelled cells in a laser-induced CNV murine model.** The animal was injected i.p. with ICG 10 days before imaging and laser CNV induced 7 days before imaging. Fifty microlitres of a CD11b antibody conjugated to fluorescein isothiocyanate (FITC) was injected into the tail vein before being imaged at 15 and 30 min post-injection to assess the identity of ICG-labelled cells. A blue-light filter (488-nm solid-state excitation laser and 500-nm barrier filter) was used for the detection of cell labelling by CD11b-FITC. A scanning laser ophthalmoscope was used to acquire all images. (A) An infrared-reflectance (IR) image (820-diode excitation laser, no barrier filter) demonstrating the presence of laser-induced CNV lesions in the deep retina/choroid. (B) A deep retinal/choroidal image obtained with a near-infrared filter (790-nm diode excitation laser and 800-nm barrier filter), showing ICG-labelled cells surrounding two laser-induced CNV lesions. (C) A magnified view of ICG-labelled cells surrounding the CNV lesion from the top lesion in (B). (D,E) A deep retinal/choroidal image of the same mouse shown in (B) at 15 min (D) and 30 min (E) after a tail vein injection of CD11b-FITC. (F) A magnified view of CD11b-FITC-labelled cells surrounding the CNV lesion from (E), showing co-labelling of most but not all ICG-labelled cells.

To test the identity of ICG^+^ cells invading the retina in the laser-induced CNV model, we used an *in vivo* staining protocol. After ICG imaging (Fig. 6A-C), a fluorescently labelled (fluorescein isothiocyanate) antibody against CD11b was injected. Over the course of 30 min, this labelled a population of cells that matched the ICG-labelled cells spatially (Fig. 6D-F). The CD11b signal was weaker than the ICG signal and there was not a perfect overlap. Nevertheless, all CD11b-positive cells were also ICG positive. The same approach was also taken with an anti-CD45 antibody, with the same outcome (data not shown). This suggests the identity of most of the ICG-labelled cells in the retina to be infiltrating myeloid cells.

## DISCUSSION

We describe here a novel technique for *in vivo* labelling of a subpopulation of circulating cells with ICG dye. We found that in order to label these cells reproducibly *in vivo*, ICG must be administered as a depot preparation. Using this approach, we showed in three disease models of retinal inflammation that infiltrating leukocytes can be detected, tracked over time and quantified in the murine retina.

We found that the commonly used i.v. route of administration of ICG failed to label infiltrating leukocytes. However, by administering ICG as a depot, we have shown that infiltrating cells were clearly labelled in our disease models. A possible explanation for this is the increase in the period of ICG exposure that circulating cells have, in order to be labelled. In keeping with this, we observed *in vitro* that an increased time of exposure to ICG resulted in increased cell labelling. Furthermore, we observed both *in vivo* and *in vitro* that the majority of labelled cells were of myeloid origin.

Our technique will be useful in the above-mentioned mouse models. In particular, the laser-induced neovascularization model is widely used to study pathological vessel growth in the retina. This model also contains an important inflammatory component, which has so far been assessed mainly by immunohistochemistry on postmortem tissue. With our ICG technique, it will be possible to monitor inflammatory cell invasion longitudinally in these animals.

More generally, in the field of inflammation, the ability to monitor a sequence of events over time is particularly important in order to establish cause-and-effect relationships. As such, recent developments have been focused on the ability to visualize inflammation *in vivo* using molecular imaging techniques, such as intravital microscopy using fluorescent antibodies, nanoparticles and transgenic animals that express fluorescent proteins (Miyahara et al., 2004; Shen et al., 2007). One study has used intravitreal ICG in murine CNV models (Paques et al., 2010). The vitreous cavity acts as a reservoir, thereby allowing prolonged exposure of cells to ICG (similar to that of a depot application). Intravitreal ICG was observed to label microglial cells for up to several weeks, allowing time-lapse observation of migratory behaviour after injury. In a similar fashion, our ICG method labels peripheral circulating cells but avoids potential neurotoxic effects of prolonged exposure of ICG to the retina, as observed by vitreoretinal surgeons who use ICG as a vital stain to visualize the internal limiting membrane during macular hole surgery (Stanescu-Segall and Jackson, 2009). Although depot ICG does not target specific cell types or epitopes like transgenic animals or antibodies, nor have we identified the identities or proportion of infiltrating cells stained, it has the advantage of having a simple application technique (depot injection), does not depend on specific mouse strains and, most importantly, has the potential for rapid translation to human use.

Current strategies for *in vivo* molecular imaging in humans include conjugation of NIR dyes, such as ICG, with ligands such as small molecules, antibodies, peptides, DNA and nanoparticles. Nevertheless, none of these probes has been approved for human use because of their so far insufficiently characterized safety profiles. In particular, nanoparticles suffer from poorly characterized distribution, accumulation and clearance from the human body, and potential cytotoxicity of heavy metal ingredients (Nel et al., 2006; Mancini et al., 2008). In contrast, ICG is an inert, water-soluble organic dye that is rapidly bound to plasma proteins and solely removed from the circulation through the liver via a specific carrier-mediated transport system (Donald and Yipintsoi, 1973). Furthermore, ICG is known not to provoke inflammation even when injected directly into tissues.

The ICG dye was first developed in the mid-1950s to determine cardiac output and hepatic function. The dye is rapidly cleared, low in toxicity and well tolerated by subjects, even at doses that exceed what is now routinely given by tenfold (Miller et al., 1962; Wood, 1962). This has led to its application to a variety of clinical uses, with examples including sentinel node biopsy, tumour demarcation surgery and lymphatic vessel assessment. The main advantage of ICG is its track record of safety and tolerance, more than 60 years of application in clinical practice, and the ability of an NIR dye to be visualized without the need for tissue windows, i.e. for non-invasive imaging.

Techniques for *in vivo* cellular imaging require a biocompatible, non-toxic agent that allows reproducible quantification of these infiltrating leukocytes. Alternative methods that have been described for *in vivo* imaging are dyes which are conjugated to antibodies, allowing more specificity of binding or *ex-vivo* labelling (labelling cells outside the circulation and reintroduction into the circulation). Our results clearly demonstrate that this simple method of using ICG as a depot injection to label cells *in vivo* can be achieved in mouse models. Translation of this insight into the clinic will involve the development of a formulation suitable for depot ICG application in humans. This might be useful not only for monitoring ocular inflammation but might also have applications further afield, for example, the detection of an immune response to new treatment strategies, such as stem cell therapies or retinal implants, or serve as an imaging biomarker for predicting the onset of angiogenesis in age-related macular degeneration or diabetic retinopathy.

## MATERIALS AND METHODS

### *In vitro* labelling of peripheral blood mononuclear cells and splenocytes with ICG

Mouse blood was drawn via cardiac puncture with a 0.5 M EDTA-coated 23-gauge needle, before either red cell lysis or Ficoll-gradient separation using Histopaque-1077 (Sigma-Aldrich, UK) according to the manufacturer's guidelines. Cells were stained with ICG, then incubated with Fc-block (BD Biosciences, UK) before primary antibody staining at the manufacturer's recommended concentrations at 4°C for 20 min. All antibodies were from BD Biosciences.

Human PBMCs were isolated from 20 ml of whole blood using a Ficoll gradient.

PBMCs were isolated from murine whole blood using Percoll-gradient separation, and splenocytes were mechanically dissociated before being incubated in 6.25 µg/ml ICG for 30 min at room temperature. PBMCs and splenocytes were washed and co-stained with CD45, CD11b and CD3 fluorescent antibodies (Miltenyi Biotech, Bisley, UK).

### Flow cytometric analysis of ICG-labelled cells

ICG-stained PBMCs, whole blood and splenocytes were analysed using a BD Bioscience LSRII flow cytometer because no commercial machine was available with a near-infrared laser for ideal excitation of ICG. Suboptimal excitation by the 633 nm red laser nonetheless resulted in a reliable signal using a 780/60 bandpass filter. A minimum of 10,000 events was collected for each sample, and fluorescence-minus-one controls were used to determine the placement of gates. Data were processed using FlowJo v10.1 (TreeStar, Ashton, OR, USA). For the toxicity assessment, gating on the following populations was performed (after exclusion of debris and cellular aggregates): lymphocytes (CD4^+^/CD8^+^/B220^+^); total myeloid populations (CD11b^+^); neutrophils (Ly6G^+^); natural killer cells (NK1.1^+^); dendritic cells (CD11c^high^); and monocytes/macrophages (CD11b^+^/CD11c^low^/Ly6G^–^/NK1.1^+^). Cell death within each population was then analysed using a live:dead stain.

### Generation of bone-marrow-derived macrophages

Generation of bone-marrow-derived macrophages (BMDMs) followed the protocol from the original report (Munder et al., 1999). In brief, mouse femurs and tibias were collected and bone marrow cells were flushed out, followed by 8 days of maturation in Teflon bags with Dulbecco's modified Eagle's medium containing 10% heat-inactivated fetal calf serum, 5% normal horse serum, 1 mM sodium pyruvate, 2 mM L-glutamine, 100 U/ml penicillin-streptomycin, 50 mM 2-mercaptoethanol (all from Life Technologies) and 100 pg/ml colony-stimulating factor 1 generated from L929 fibroblast-conditioned media.

### Mouse interleukin-6 ELISA

BMDMs were plated at a density of 5×10^5^ cells/well in a 96-well flat-bottomed plate. Four hours later, the medium was exchanged for 0.2 mg/ml ICG dye, with either medium alone or 5 ng/ml LPS. After the indicated times, supernatants were collected and frozen at −80°C. Mouse IL-6 sandwich ELISA was performed in technical triplicate according to the manufacturer's instructions (BD Biosciences, UK) using rat anti-mouse IL-6, 554400 and biotin rat anti-mouse IL-6, 554402.

### Spectrophotometry of ICG

BMDMs were plated in flat-bottomed 96-well plates for 4 h in 10% fetal calf serum and Dulbecco's modified Eagle's medium, before the addition of ICG to a concentration of 0.2 mg/ml. Cells were incubated for the specified amount of time before washing five times with PBS. After this, 200 μl of PBS was added to the well for 5 min and then removed for spectrophotometry. Triton (2%) in 200 μl PBS was then added to each well and the supernatant also removed after 5 min. Spectrophotometry was performed using a SpectraMax 190 (Molecular Devices, Wokingham, UK), with SoftMax Pro (v6.0) software, set to an absorbance of 790 nm.

### Bright-field microscopy

BMDMs were imaged in flat-bottomed 96-well plates with a Leica DMIRB microscope using Leica QFluoro software v3.1. No filters were used, but light intensity was adjusted.

### Magnetic-activated cell sorted separation of CD4^+^ cells

Single-cell spleen suspensions from C57BL/6 mice were erythrocyte lysed by ammonium-calcium-potassium (ACK) buffer. Splenic CD4 cells were prepared by enriching CD4^+^ cells using anti-CD4 microbeads (Miltenyi Biotech, UK) according to the manufacturer's instructions.

### Animals

All animals were handled in accordance with the UK Animals (Scientific Procedures) Act 1986. Female C57BL/6J mice (Harlan, UK) at 7-8 weeks of age were used. For *in vivo* procedures, the mice were anaesthetized with an i.p. injection of medetomindine hydrochloride (1 mg/kg body weight; Domitor; Pfizer Animal Health, New York, NY, USA) and ketamine (60 mg/kg body weight) in water. Pupillary dilatation was achieved with one drop of 1% Tropicamide (Bausch and Lomb, Kingston-upon-Thames, UK).

### Induction of endotoxin-induced uveitis

Female C57BL/6J mice (Harlan, UK) at 8 weeks of age received a single i.p. injection of 0.2 mg of LPS from *E. coli* (Sigma-Aldrich, St Louis, MO, USA) in PBS. A 1 mg i.p. bolus injection of ICG was given 24 h before the LPS treatment. Imaging was performed 48 h later, at a previously determined time point when there is peak infiltration by myeloid cells.

### Induction of experimental autoimmune uveoretinitis

Female C57BL/6J mice (Harlan, UK) at 7 weeks of age received 500 μg of human RBP-1-20 peptide subcutaneously, emulsified in complete Freund's adjuvant (Sigma-Aldrich, UK) supplemented with 1.5 mg/ml *Mycobacterium tuberculosis* H37RA (Difco Laboratories, BD, Oxford, UK). Pertussis toxin (1.5 μg) was simultaneously administered into the peritoneal space (Tocris Bioscience, Bristol, UK). Imaging was performed 26 days later at the time point determined to be the disease peak in our facility using this protocol.

### Induction of laser choroidal neovascularization

In female C57BL/6J mice (Harlan, UK) at 7-8 weeks of age, laser CNV was induced using a slit-lamp-mounted diode laser system (wavelength 680 nm; Keeler, Windsor, UK). The laser settings used were as follows: 200 mW power, 100 ms duration and 100 µm spot diameter. Laser CNV lesions were applied at a distance of three disc diameters from the optic nerve, avoiding any blood vessels.

### *In vivo* imaging

Ocular imaging was performed using a scanning laser ophthalmoscope (Spectralis™ HRA; Heidelberg Engineering, Heidelberg, Germany). A lens with a 55° field of view was used, and a mean of 100 consecutive frames was taken for each image. In order to visualize ICG-labelled cells, a near-infrared filter (790 nm diode excitation laser and 800 nm long-pass filter) was used. Cell labelling was achieved with various doses (from 1 to 0.0125 mg; Fig. S4) of 5 mg ICG (Pulsion Medical Systems AG) dissolved in 5 ml of water and administered 3 days before laser induction of CNV, i.p. injection of LPS or imaging of the experimental autoimmune uveoretinitis model. The different routes of administration are summarized in Fig. S5. To assist the identification of laser CNV lesions, infrared-reflectance imaging was performed with an 820 diode excitation laser and no barrier filter. For autofluorescence imaging and fluorescein angiography, a blue-light filter (488 nm solid-state excitation laser and 500 nm long-pass filter) was used. Fluorescein angiography was performed 1 week after laser CNV induction with an i.p. injection of 0.2 ml fluorescein sodium (2%). Images were acquired at 90 s and 7 min after injection.

### Quantification of ICG^+^ cells in the retina

Images were exported from the Heidelberg eye explorer version 1.7.1.0 and processed in Adobe Photoshop CS5 (Adobe Systems, San Jose, CA, USA). Details of image processing and quantification can be found in the Results section and Fig. S5.

## References

[DMM019018C1] Boldison J., Chu C. J., Copland D. A., Lait P. J. P., Khera T. K., Dick A. D. and Nicholson L. B. (2014). Tissue-resident exhausted effector memory CD8+ T cells accumulate in the retina during chronic experimental autoimmune uveoretinitis. *J. Immunol.* 192, 4541-4550. 10.4049/jimmunol.1301390 24740509 PMC4009498

[DMM019018C2] Cherrick G. R., Stein S. W., Leevy C. M. and Davidson C. S. (1960). Indocyanine green: observations on its physical properties, plasma decay, and hepatic extraction. *J. Clin. Invest.* 39, 592-600. 10.1172/JCI104072 13809697 PMC293343

[DMM019018C3] Chu C. J., Herrmann P., Carvalho L. S., Liyanage S. E., Bainbridge J. W. B., Ali R. R., Dick A. D. and Luhmann U. F. O. (2013). Assessment and in vivo scoring of murine experimental autoimmune uveoretinitis using optical coherence tomography. *PLoS ONE* 8, e63002 10.1371/journal.pone.0063002 23690973 PMC3653962

[DMM019018C4] Cibiel A., Pestourie C. and Duconge F. (2012). In vivo uses of aptamers selected against cell surface biomarkers for therapy and molecular imaging. *Biochimie* 94, 1595-1606. 10.1016/j.biochi.2012.02.025 22738730

[DMM019018C5] Dick A. D., Ford A. L., Forrester J. V. and Sedgwick J. D. (1995). Flow cytometric identification of a minority population of MHC class II positive cells in the normal rat retina distinct from CD45lowCD11b/c+CD4low parenchymal microglia. *Br. J. Ophthalmol.* 79, 834-840. 10.1136/bjo.79.9.834 7488603 PMC505270

[DMM019018C6] Dick A. D., McMenamin P. G., Korner H., Scallon B. J., Ghrayeb J., Forrester J. V. and Sedgwick J. D. (1996). Inhibition of tumor necrosis factor activity minimizes target organ damage in experimental autoimmune uveoretinitis despite quantitatively normal activated T cell traffic to the retina. *Eur. J. Immunol.* 26, 1018-1025. 10.1002/eji.1830260510 8647162

[DMM019018C7] Donald D. E. and Yipintsoi T. (1973). Comparison of measured and indocyanine green blood flows in various organs and systems. *Mayo Clin. Proc.* 48, 492-500. 4577311

[DMM019018C8] Horie S., Robbie S. J., Liu J., Wu W.-K., Ali R. R., Bainbridge J. W., Nicholson L. B., Mochizuki M., Dick A. D. and Copland D. A. (2013). CD200R signaling inhibits pro-angiogenic gene expression by macrophages and suppresses choroidal neovascularization. *Sci. Rep.* 3, 3072 10.1038/srep03072 24170042 PMC3812658

[DMM019018C9] Hossain P., Liversidge J., Cree M. J., Manivannan A., Vieira P., Sharp P. F., Brown G. C. and Forrester J. V. (1998). In vivo cell tracking by scanning laser ophthalmoscopy: quantification of leukocyte kinetics. *Invest. Ophthalmol. Vis. Sci* 39, 1879-1887. 9727411

[DMM019018C10] Joussen A. M., Murata T., Tsujikawa A., Kirchhof B., Bursell S.-E. and Adamis A. P. (2001). Leukocyte-mediated endothelial cell injury and death in the diabetic retina. *Am. J. Pathol.* 158, 147-152. 10.1016/S0002-9440(10)63952-1 11141487 PMC1850259

[DMM019018C11] Kogure K., David N. J., Yamanouchi U. and Choromokos E. (1970). Infrared absorption angiography of the fundus circulation. *Arch. Ophthalmol.* 83, 209-214. 10.1001/archopht.1970.00990030211015 4983539

[DMM019018C12] Lee R. W., Nicholson L. B., Sen H. N., Chan C.-C., Wei L., Nussenblatt R. B. and Dick A. D. (2014). Autoimmune and autoinflammatory mechanisms in uveitis. *Semin. Immunopathol.* 36, 581-594. 10.1007/s00281-014-0433-9 24858699 PMC4186974

[DMM019018C13] Liu J., Copland D. A., Horie S., Wu W.-K., Chen M., Xu Y., Paul Morgan B., Mack M., Xu H., Nicholson L. B. et al. (2013). Myeloid cells expressing VEGF and arginase-1 following uptake of damaged retinal pigment epithelium suggests potential mechanism that drives the onset of choroidal angiogenesis in mice. *PLoS ONE* 8, e72935 10.1371/journal.pone.0072935 23977372 PMC3745388

[DMM019018C14] Lopez P. F., Grossniklaus H. E., Lambert H. M., Aaberg T. M., Capone A. Jr, Sternberg P. Jr and L'Hernault N. (1991). Pathologic features of surgically excised subretinal neovascular membranes in age-related macular degeneration. *Am. J. Ophthalmol.* 112, 647-656. 10.1016/S0002-9394(14)77270-8 1957899

[DMM019018C15] Lutty G. A., Cao J. and McLeod D. S. (1997). Relationship of polymorphonuclear leukocytes to capillary dropout in the human diabetic choroid. *Am. J. Pathol.* 151, 707-714. 9284819 PMC1857840

[DMM019018C16] Mancini M. C., Kairdolf B. A., Smith A. M. and Nie S. (2008). Oxidative quenching and degradation of polymer-encapsulated quantum dots: new insights into the long-term fate and toxicity of nanocrystals in vivo. *J. Am. Chem. Soc.* 130, 10836-10837. 10.1021/ja8040477 18652463 PMC3743542

[DMM019018C17] Miller D. E., Gleason W. L. and Mc I. H. (1962). A comparison of the cardiac output determination by the direct Fick method and the dye-dilution method using indocyanine green dye and a cuvette densitometer. *J. Lab. Clin. Med.* 59, 345-350. 14473967

[DMM019018C18] Miyahara S., Kiryu J., Miyamoto K., Katsuta H., Hirose F., Tamura H., Musashi K., Honda Y. and Yoshimura N. (2004). In vivo three-dimensional evaluation of leukocyte behavior in retinal microcirculation of mice. *Invest. Ophthalmol. Vis. Sci.* 45, 4197-4201. 10.1167/iovs.04-0192 15505075

[DMM019018C19] Montet-Abou K., Daire J.-L., Hyacinthe J.-N., Jorge-Costa M., Grosdemange K., Mach F., Petri-Fink A., Hofmann H., Morel D. R., Vallee J.-P. et al. (2010). In vivo labelling of resting monocytes in the reticuloendothelial system with fluorescent iron oxide nanoparticles prior to injury reveals that they are mobilized to infarcted myocardium. *Eur. Heart J.* 31, 1410-1420. 10.1093/eurheartj/ehp547 20023288

[DMM019018C20] Montezuma S. R., Vavvas D. and Miller J. W. (2009). Review of the ocular angiogenesis animal models. *Semin. Ophthalmol.* 24, 52-61. 10.1080/08820530902800017 19373687

[DMM019018C21] Munder M., Eichmann K., Moran J. M., Centeno F., Soler G. and Modolell M. (1999). Th1/Th2-regulated expression of arginase isoforms in murine macrophages and dendritic cells. *J. Immunol.* 163, 3771-3777. 10490974

[DMM019018C22] Nahrendorf M., Sosnovik D. E., Waterman P., Swirski F. K., Pande A. N., Aikawa E., Figueiredo J.-L., Pittet M. J. and Weissleder R. (2007). Dual channel optical tomographic imaging of leukocyte recruitment and protease activity in the healing myocardial infarct. *Circ. Res.* 100, 1218-1225. 10.1161/01.RES.0000265064.46075.31 17379832

[DMM019018C23] Nel A., Xia T., Madler L. and Li N. (2006). Toxic potential of materials at the nanolevel. *Science* 311, 622-627. 10.1126/science.1114397 16456071

[DMM019018C24] Paques M., Simonutti M., Augustin S., Goupille O., El Mathari B. and Sahel J.-A. (2010). In vivo observation of the locomotion of microglial cells in the retina. *Glia* 58, 1663-1668. 10.1002/glia.21037 20578032

[DMM019018C25] Penfold P. L., Provis J. M. and Billson F. A. (1987). Age-related macular degeneration: ultrastructural studies of the relationship of leucocytes to angiogenesis. *Graefes Arch. Clin. Exp. Ophthalmol.* 225, 70-76. 10.1007/BF02155808 2436980

[DMM019018C26] Rausch M., Sauter A., Frohlich J., Neubacher U., Radu E. W. and Rudin M. (2001). Dynamic patterns of USPIO enhancement can be observed in macrophages after ischemic brain damage. *Magn. Reson. Med.* 46, 1018-1022. 10.1002/mrm.1290 11675656

[DMM019018C27] Roivainen A., Jalkanen S. and Nanni C. (2012). Gallium-labelled peptides for imaging of inflammation. *Eur. J. Nucl. Med. Mol. Imaging* 39 Suppl. 1, 68-77. 10.1007/s00259-011-1987-6 22388620

[DMM019018C28] Shen J., Xie B., Dong A., Swaim M., Hackett S. F. and Campochiaro P. A. (2007). In vivo immunostaining demonstrates macrophages associate with growing and regressing vessels. *Invest. Ophthalmol. Vis. Sci.* 48, 4335-4341. 10.1167/iovs.07-0113 17724225

[DMM019018C29] Stanescu-Segall D. and Jackson T. L. (2009). Vital staining with indocyanine green: a review of the clinical and experimental studies relating to safety. *Eye* 23, 504-518. 10.1038/eye.2008.249 18670454

[DMM019018C30] van Hemert F. J., Thurlings R., Dohmen S. E., Voermans C., Tak P. P., van Eck-Smit B. L. F. and Bennink R. J. (2007). Labeling of autologous monocytes with 99mTc-HMPAO at very high specific radioactivity. *Nucl. Med. Biol.* 34, 933-938. 10.1016/j.nucmedbio.2007.07.008 17998095

[DMM019018C31] Whitcup S. M., Sodhi A., Atkinson J. P., Holers V. M., Sinha D., Rohrer B. and Dick A. D. (2013). The role of the immune response in age-related macular degeneration. *Int. J. Inflam.* 2013, 348092 10.1155/2013/348092 23762772 PMC3676958

[DMM019018C32] Wood E. H. (1962). Diagnostic applications of indicator-dilution technics in congenital heart disease. *Circ. Res.* 10, 531-568. 10.1161/01.RES.10.3.531 14008164

[DMM019018C33] Xu H., Chen M. and Forrester J. V. (2009). Para-inflammation in the aging retina. *Prog. Retin. Eye Res.* 28, 348-368. 10.1016/j.preteyeres.2009.06.001 19560552

